# Who Gets Credit for AI-Generated Art?

**DOI:** 10.1016/j.isci.2020.101515

**Published:** 2020-08-29

**Authors:** Ziv Epstein, Sydney Levine, David G. Rand, Iyad Rahwan

**Affiliations:** 1 MIT Media Lab, 75 Amherst Street, Cambridge, MA 02139, USA; 2 Department of Brain and Cognitive Sciences, 43 Vassar Street, Cambridge, MA 02139, USA; 3 Department of Psychology, Harvard University, 33 Kirkland Street, Cambridge, MA 02139, USA; 4 Sloan School of Management, MIT, 100 Main Street, Cambridge, MA 02139, USA; 5 Center for Humans & Machines, Max Planck Institute for Human Development, Lentzeallee 94, 14195 Berlin, Germany

**Keywords:** Computer Science, Artificial Intelligence, Economics

## Abstract

The recent sale of an artificial intelligence (AI)-generated portrait for $432,000 at Christie's art auction has raised questions about how credit and responsibility should be allocated to individuals involved and how the anthropomorphic perception of the AI system contributed to the artwork's success. Here, we identify natural heterogeneity in the extent to which different people perceive AI as anthropomorphic. We find that differences in the perception of AI anthropomorphicity are associated with different allocations of responsibility to the AI system and credit to different stakeholders involved in art production. We then show that perceptions of AI anthropomorphicity can be manipulated by changing the language used to talk about AI—as a tool versus agent—with consequences for artists and AI practitioners. Our findings shed light on what is at stake when we anthropomorphize AI systems and offer an empirical lens to reason about how to allocate credit and responsibility to human stakeholders.

## Highlights

• There is variation in the extent to which people perceive AI as anthropomorphic
• Perception of AI anthropomorphicity is related to allocation of responsibility
• Perception of AI anthropomorphicity can be manipulated by changing language
• We must be careful with our words when talking about AI

## Graphical Abstract

**Figure:**
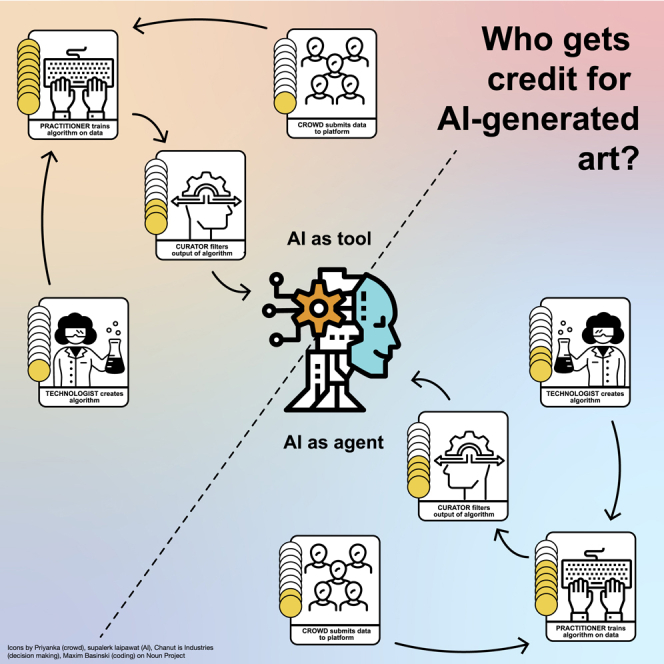

## Introduction

**Figure 1:**
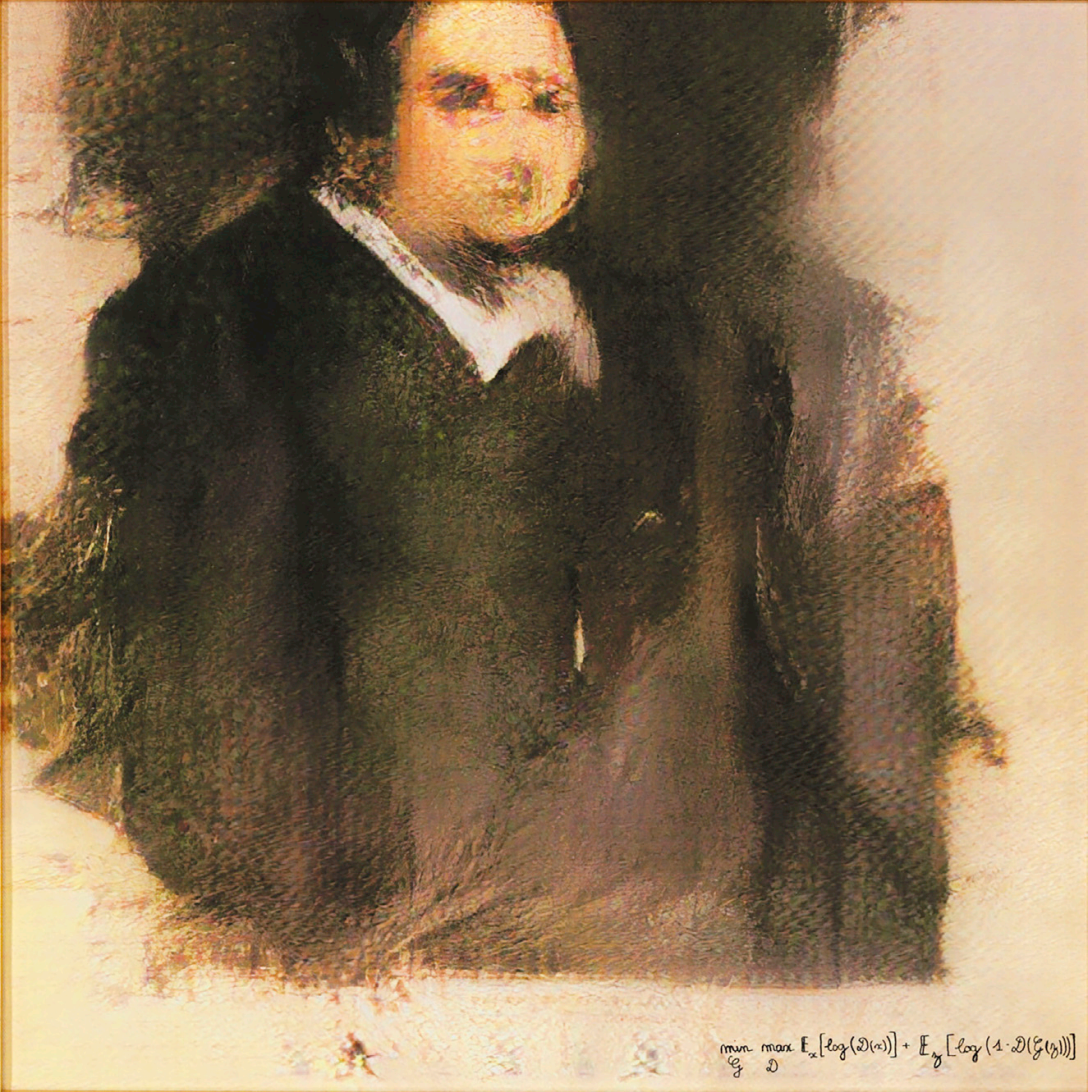
Image of the Painting Edmond de Belamy, which Sold for $432,500 at Christie's Art Auction

On October 25, 2018, a portrait generated by a machine learning (ML) algorithm called a generative adversarial network (or GAN) (Goodfellow et al., 2014) sold at Christie's art auction for $432,500. As Christie's initial estimate for the piece was $10,000, its sale for over 40 times this expectation shocked the art world. Marketed by Christie's as “the first portrait generated by an algorithm to come up for auction,’’ the painting—entitled *Edmond De Belamy* (see Figure 1)—struck a chord about the nature of authorship and artificial intelligence (AI) (Cohn, 2018).

Yet the reality of the painting's creation is not as clear as Christie's purports. Even though AI played a role in generating the artwork, *Edmond de Belamy* would never have been produced without the help of humans. It was the Parisian art collective Obvious who selected, printed, marketed, and sold the image; but the human involvement does not stop there. The algorithm was trained on the paintings of Renaissance masters, sourced from WikiArt. Ian Goodfellow invented the original GAN architecture, and Alec Radford, Luke Metz, and Soumith Chintala innovated the DCGAN that actually generated the artwork. But perhaps the most relevant here is the then-19-year-old artist and technologist, Robbie Barrat, who wrote code to produce Renaissance-style images with DCGAN (Learn more about his GitHub repo here: https://github.com/robbiebarrat/art-DCGAN) and which was ostensibly lightly repurposed to produce *Edmond* *de Belamy*. Barrat noted that Obvious “almost immediately started producing work identical to the outputs of the pre-trained portrait and landscape networks” he had put online (Vincent, 2018). Neither Barrat nor the ML researchers received any of the $432,500, which all went to Obvious.

Although the humans involved in the creation of *Edmond de Belamy* were essentially cut out of the art's creation narrative, the AI itself was often spoken about as having human-like characteristics. In a press release, Obvious told reporters that “an artificial intelligence managed to create art,” which underpinned their motto that “creativity isn't only for humans.” When Christie's was raising awareness about the impending auction of *Edmond De Belamy*, they also employed anthropomorphic language to increase hype for the work: “This portrait … is not the product of a human mind. It was created by an artificial intelligence, an algorithm defined by that algebraic formula with its many parentheses” (Anonymous, 2018). Another spokesperson went further saying, “We are offering a public platform to exhibit an artwork that has entirely been realised by an algorithm,” (Hitti, 2018). The media ran with this narrative, creating a discourse that emphasized the autonomy and agency of the algorithm (Table 1 contains further examples.).

**Table 1:** Media Snippets from the Edmond de Belamy Case

Quote	Source
This portrait … is not the product of a human mind. **It was created by an artificial intelligence**, an algorithm defined by that algebraic formula with its many parentheses	Christie's (Anonymous, 2018)
AI has already been incorporated as a tool by contemporary artists and as this technology further develops, we are excited to participate in these continued conversations. To best engage in the dialogue, we are offering a public platform to exhibit an artwork that has entirely been realised by an algorithm,	Christie's (Hitti, 2018)
Christie's, the auction house that has sold paintings by picasso and monet at record prices, was poised on Tuesday to set another milestone with **the first-ever auction of art created by artificial intelligence.**	Reuters (Goldberg, 2018)
The painting, titled “the portrait of edmond belamy,” **was completed by artificial intelligence managed by a Paris-based collective called Obvious,** Christie's said.	USA Today (Molina, 2018)
Whether art or not, the signature of the ‘artist’ at the bottom of the painting gives away **its origin as a product of machine learning rather than human hand.**	PC Mag (Smith, 2018)
Once the software “**understood** the rules of portraiture” using a new algorithm developed by Google researcher Ian Goodfellow, it then generated a series of new images **by itself**, Fautrel said.	NDTV (France-Presse, 2018)

Agentic language is bolded.

The story of *Edmond de Belamy* underscores two general obstacles for the accountability and governance of AI systems, which are critical to understanding the complexity of assigning credit and responsibility in AI art cases. The first obstacle is knowing what the set of possibly relevant human stakeholders are and how they are relatively positioned within an AI system. Indeed, AI is a diffuse term that corresponds to a web of human actors and computational processes interacting in complex ways (Seaver, 2017). This complexity may lead to situations wherein individual responsibility and accountability is obfuscated due to a lack of clear understanding of who the relevant actors are and how they interact. Such lack of understanding can manifest as the Moral Crumple Zone, whereby disproportional outrage is channeled toward a peripheral person of an AI system simply because the person is closest to the transgression (think about an upset customer yelling at the employee at the flight kiosk when their flight is canceled, despite the fact that the employee had nothing to do with the cancellation itself) (Elish, 2019). Our intuitive moral understanding of actors and transgressions may be at odds with the inherent complexity of AI systems.

Previous studies of the social impact of AI have considered a wide range of possible human stakeholders. In the context of autonomous vehicles (AVs), Waytz et al. consider the human passenger, the car itself, the people who designed the car, and the company that developed the car (Waytz et al., 2014), whereas Awad and Levine et al. consider the human passenger, the car itself, the company who created it, and the programmer who implemented the car's software (Awad et al., 2018). In the context of AI art, Eshraghian distinguishes between the programmer, the trainer, and the user (Eshraghian, 2020), whereas McCormack et al. similarly distinguish between the creators of the software, curators of datasets, and those who train the algorithm and modify parameters (McCormack et al., 2019).

A second obstacle is the phenomenon of anthropomorphizing AI systems. With the recent boom of suprahuman performance on such tasks as Atari games (Mnih et al., 2015), Go (Silver et al., 2016), and lung cancer detection (Ardila et al., 2019), we have seen a proliferation of the anthropomorphization of AI in the media (Proudfoot, 2011; Watson, 2019; Salles et al., 2020). This has been exacerbated by the ML literature itself (Lipton and Steinhardt, 2018), where many ML tasks and techniques are described using the same language we would use for a human doing the task—sreading comprehension (Hermann et al., 2015), music composition (Mozer, 1994), curiosity (Schmidhuber, 1991), fear (Lipton et al., 2016), “thought” vectors (Kiros et al., 2015), and “consciousness” priors (Bengio, 2017).

But what is at stake when we anthropomorphize AI? Recent work reveals how anthropomorphization can affect trust. Through a series of experiments involving an unavoidable crash in a driving simulator with cars of varying complexity (i.e., a normal car versus a self-driving car versus an anthropomorphized self-driving car with a human voice and name), Waytz et al. show that increases in the anthropomophization of a car predicts trust in the car (Waytz et al., 2014). Although they mostly focused on the psychological construct of trust, they also found that anthropomorphization affects attributions of responsibility and punishment for the car's mistakes, which is consistent with the established relationship between the agency and perceived responsibility (Epley et al., 2007; Waytz et al., 2014). This builds on a growing body of work that our “mind perception” (which manifests as inferences of intentions, beliefs, and values) meaningfully varies across individuals and shapes our moral judgments (Epley et al., 2007; Gray et al., 2007, 2012; Waytz et al., 2010).

There is also the concern that anthropomorphizing AI systems can “undermine our ability to hold powerful individuals and groups accountable for their technologically-mediated actions” (Watson, 2019). When an AI system causes a moral transgression, it may be the case that the programmer or systems architect can eschew personal responsibility by blaming the “unexpected behavior” of the system, downplaying their own involvement. Along these lines in the context of AVs, Gill found that participants thought harming a pedestrian was more permissible for an AV when compared with a human in a regular car and that the attribution of responsibility to the AV drove the shift in moral judgment (Gill, 2020).

Ultimately, as AI systems become further integrated into human decision-making, it is likely that they will be increasingly anthropomorphized. Thus, understanding the psychological mechanics of this “absorption of responsibility” by the AI is important for the accountability and governance of AI systems. In particular, in line with Watson, one might expect that increased anthropomorphicity of an AI system may diminish the perceived responsibility of all human actors involved (Watson, 2019). Yet ultimately this is an empirical question subject to inquiry.

In this article, we use the case of *Edmond de Belamy* to explore these questions in the context of AI art: not only was there ambiguity about the humans involved in the creation process but also rampant anthropormorphizaton of the process itself.

To those ends, we focus on two main research questions:

1. How do people think credit and responsibility should be allocated to various actors in the production of AI art?
2. How do these intuitions vary based on people's perceptions of the anthropomorphicity of the AI system?

These research questions are closely related to, but distinct from, the broader philosophical questions related to AI art, such as “Can computers create art?” Hertzmann traces the histories of several art automation technologies (such as the camera and animation) to argue that generative AI technologies are yet another artistic tool, with their own distinct affordances (Hertzmann, 2018). As such, he contends that art is necessarily authored by social agents, and thus AI algorithms (as understood today) cannot be credited with authorship of art. McCormack et al. build on these ideas in the context of the *Edmond de Belamy* phenomenon (McCormack et al., 2019). They conclude that “The creator of the software and person who trained and modified parameters to produce the work can both be considered authors,” but that “AI systems are not broadly accepted as authors by artistic or general public communities.”

These scholars make convincing arguments for why AI systems *ought not* be credited with authorship. Our investigation concerns a different question, namely, how *does* the public assign credit to an AI involved in making art? In particular, we use a series of vignette studies to directly explore the relationship between anthropomorphicity of the AI and the levels of responsibility assigned to various actors in an AI system. By focusing on peoples' intuitions in these vignettes, we consider credit and responsibility in the broad sense of public perception, rather than in the legal or prescriptive sense (Colton, 2008; Eshraghian, 2020).

### The Terminology of AI Art

Computer-generated artwork has a long and diverse history and involves a wide range of AI tools and AI-human interaction paradigms. Some use interactive evolutionary algorithms to crowd-source the creation and curation of artifacts (Draves, 2005; Epstein et al., 2020; Secretan et al., 2011; Sims, 1991), whereas others have created platforms for artists and practitioners to use AI models, such as RunwayML, GANPaint (Bau et al., 2018), and DeepAngel (Groh et al., 2019). In addition to GANs, many other visual generative algorithms have been explored, such as neural style transfer (Gatys et al., 2016), Computational Aesthetics (Machado et al., 2008), Fractal Flame (Draves, 2005; Draves and Reckase, 2003), deep learning-powered adversarial evolution (Blair, 2019), and hybrid methods (Colton, 2008, 2012). Here, following the case study of *Edmond de Belamy*, we trace a particular type of AI art, where the system is presented with human artwork and attempts to mimic the style of the human artists. This process involves both a specific AI technology (e.g., the GAN) and a corresponding workflow, which inspired our vignettes (described in full in Tables S1 and S2).

## Results

### Study 1

In Study 1, participants read a stylized vignette that described the process by which AI artwork is created. They were asked to allocate responsibility and monetary credit to the agents involved in the creation of the AI art. Then, they were asked four questions designed to elicit their perception of the AI's anthropomorphicity (Waytz et al., 2014), which were combined into an aggregate score (for more information on the vignette-dependent variables and anthropomorphicity measure, see Supplementary Information). We hypothesized that participants who anthropomorphize the AI system to a greater extent will allocate more responsibility to the AI system itself. In addition, subjects were randomly assigned to one of two conditions. In one condition, the art is found to violate copyright law and a fine is levied against it (negative outcome). In the other condition, the art received positive reception and is sold at a prestigious auction house (positive outcome).

**Figure 2:**
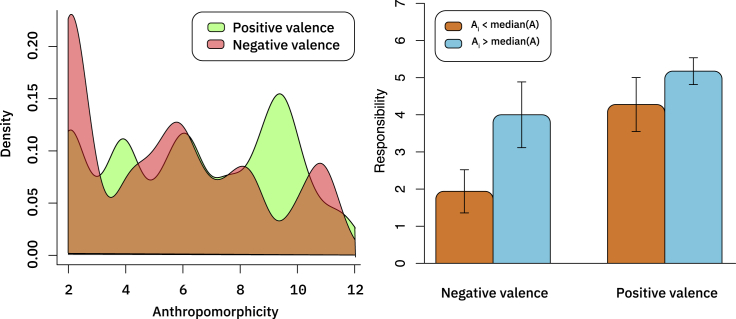
Anthropomorphicity and Repsonsibility by Valence Left: kernel density plot of anthropomorphicity measure for positive valence (art received positive reception and is sold at a prestigious auction house, in green) and negative valence (the art is found to violate copyright law and a fine is levied against it, in red) outcomes. Brown is overlap between the two. Right: Responsibility allocated to the AI system for users who perceive the system as anthropomorphic above and below the median, by valence. Means with 95% confidence intervals.

For both the positive and negative valence conditions, we see substantial variation in AI anthropomorphicity (see the left pane of Figure 2). This indicates that different participants had markedly different baseline perceptions of AI.

We now turn to assessing the impact of these differences in perception on attribution of responsibility. Following our pre-registered analysis plan, we collapse across valence conditions and see that participants who anthropomorphize the AI more also assign more responsibility to it: participants who rated the system more than the median anthropomorphicity score assigned significantly more responsibility (4.75) to the AI than the participants who rated the system less than the median anthropomorphicity (4.75 versus 3.03, respectively, *t* = −5.1159, *df* = 113.67, p < 0.001, preregistered).

In a follow-up *post hoc* analysis, we enter the data into a regression model to see whether anthropomorphicity (using the continuous measure) and valence interact. We find a significant main effect of anthropomorphicity (*t* = 4.634, p < 0.0001) as well as valence (t = 4.816, p < 0.0001), and we also find a significant positive interaction between them (*t* = −2.295, p = 0.0234). Decomposing this interactions shows that whereas there is at least a marginally significant positive relationship between anthropomorphicity and AI responsibility in both valence conditions, the relationship is significantly stronger in the negative valence condition (*r* = 0.4994, *t* = 4.237, p < 0.0001) relative to the positive valence condition (*r* = 0.2111, *t* = 1.794, p = 0.0771).

These findings suggest that the extent to which people perceive the AI system as an agent is correlated with the extent to which they allocate responsibility to it, extending results from prior work on the AVs (Waytz et al., 2010) to the context of art. But how does this impact the responsibility of the other actors involved in the production of AI art? Critically, the *Edmond de Belamy* case suggests that the mind perception of the AI system impacts how people assign responsibility not only to the system itself but also to proximal humans (such as Obvious or Robbie Barrat).

**Figure 3:**
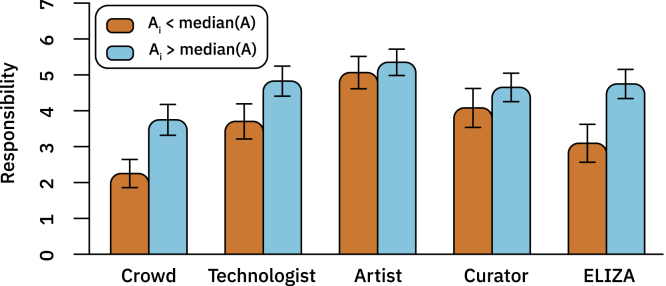
Allocation of Responsibility to Each of the Actors Involved in the Creation of AI-Generated Art, Collapsed Across Valence The roles of crowd, technologist, artist, curator and ELIZA are described in Table S1. Means with 95% confidence intervals.

Therefore, in addition to looking at the attributions of responsibility to the AI system itself, we also consider various involved human actors, such as the artist (i.e., the person taking the inputs and the learning algorithms and producing a trained algorithm), the curator (i.e., the person who selects the final artwork and brings it to auction), the technologist (i.e., the person who creates the learning algorithm), and the crowd (i.e., the people whose labor is responsible for creating the inputs to the algorithm), as shown in Figure 3. We find that participants who anthropomorphized the AI more than the median assign more responsibility to the crowd and technologist, when compared with those who anthropomorphized the AI less than the median (*t* = 5.3214, p < 0.0001 and *t* = 3.5603, p = 0.00026 for crowd and technologist, respectively). We also observed a marginal increase in responsibility assigned to the curator (*t* = 1.6227, p = 0.05374) and no change in responsibility assigned to the artist (*t* = 1.0138, p = 0.1564). As a result, participants who anthropomorphized the AI more assigned less proportional credit to the artist (as they assigned more responsibility to other roles, and not any more responsibility to the artist).

### Study 2

In Study 2, we test whether the correlational relationships observed in Study 1 are in fact causal. We do so by experimentally manipulating the perceived anthropomorphicity of the AI, and considering the impact of that manipulation on perception of the *humans* involved. As in Study 1, participants read a vignette that described the process of AI art creation. In the Tool Condition, the AI was described as a tool used by a human artist. In the Agent Condition, the AI was described as an agentic and anthropomorphized AI artist (see Supplementary Information for vigenettes). By directly manipulating the anthropormorphicity of the AI system (conceptually following the approach of Malle et al. (2016) from the field of Human Robot Interaction), we can causally assess the impact of anthropomorphization.

**Figure 4:**
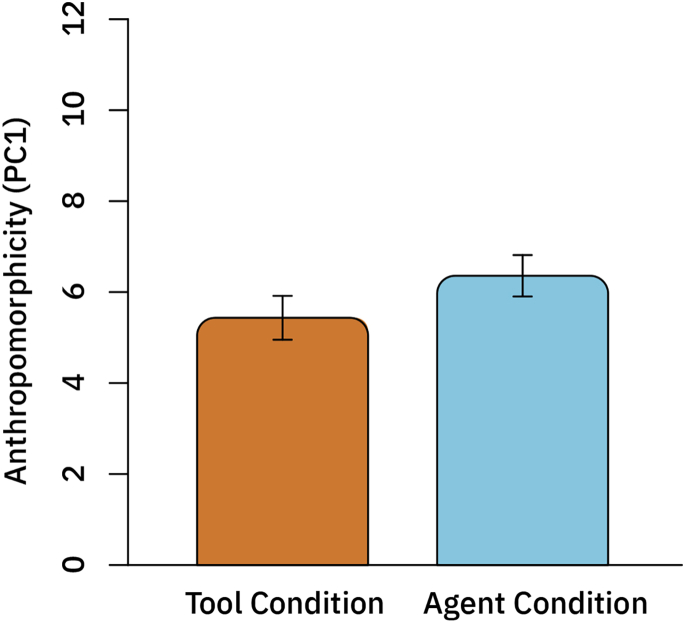
Anthropomorphicity (e.g., First Principal Component of the Principal-Component Analysis) for the Condition Describing the AI as a Tool and as an Agent, Respectively Means with 95% confidence intervals.

As anticipated, we find a significant difference in perceived anthropomorphicity of the AI agent by condition, as shown in Figure 4 (*t* = −2.75, *df* = 317.99, p = 0.003). This manipulation check indicates that our treatments were successful in affecting participants' conceptualizations of the AI's anthropomorphicity.

**Figure 5:**
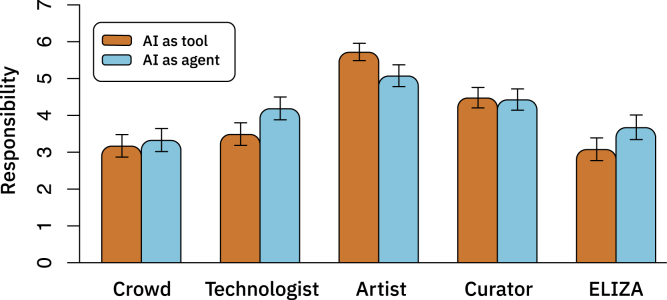
Allocation of Responsibility to Each of the Actors Involved in the Creation of AI-Generated Art The roles of crowd, technologist, artist, curator, and ELIZA are described in Table S2. Means with 95% confidence intervals.

Consistent with the correlational results in Study 1, we find that when the AI system is described as an agent, participants ascribe more responsibility it, compared with when the AI system is described as a non-agent (*t* = 2.5928, *df* = 311.69, p = 0.0004, pre-registered; Figure 5). We also find that participants ascribe less responsibility to the artist who used the AI system in the agentic condition, when compared with when the AI system is described as a non-agent (*t* = −3.375, *df* = 293.05, p = 0.0004). In contrast, participants ascribe more responsibility to the technologist who used the AI system in the agentic condition, when compared with when the AI system is described as a non-agent (*t* = 3.158, *df* = 316.35, p = 0.0008).

We find these results are robust to control for valence. For responsibility to the AI, we find a main effect for both the agent treatment (p = 0.00746) and valence (p < 0.0001). For responsibility to the technologist, we find a main effect for both the agent treatment (p *=* 0.00542) and valence (p < 0.0001). For responsibility to the artist, we find a main effect for both the agent treatment (p = 0.02728) and valence (p = 0.00581). In none of these cases did we find an interaction effect between valence and agent (p = 0.3422 for AI, p = 0.64415 for artist, and p = 0.53880 for technologist).

For responsibility to the crowd, we find a marginal effect for the agent treatment (p = 0.0783), a significant effect for valence (p < 0.0001), and a marginal interaction effect (p = 0.0627). For responsibility to the curator, we find no effect for the agent treatment (p = 0.261), a marginal effect for valence (p = 0.101), and a marginal interaction effect (p = 0.059).

**Figure 6:**
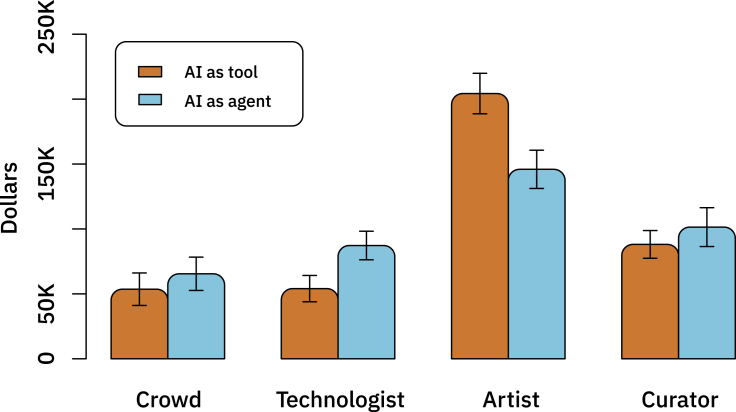
Allocation of Dollars to Each of the Actors Involved in the Creation of the AI-Generated Art (Out of Total Sum of $400K) Means with 95% confidence intervals.

Subjects were also asked to assign credit (in the form of monetary awards or fines) to each of the humans in the system. Results mirror those of the responsibility judgments, although there is more variance in the dollar allocation (Figure 6). When the AI system is described as an agent, participants ascribe less fine/award to the artist (t = −5.37, df = 317.99, p value = <0.0001, pre-registered), and more fine/award to the technologist who developed the AI system, when compared with when the AI is described as a non-agent (t = −4.38, df = 311.43, p value = <0.0001). Conversely, we found no significant difference across conditions in the fine/award ascribed to the crowd (p = 0.273), but a marginally significant difference across conditions in the fine/award ascribed to the curator (p = 0.07654). We also find no interactions between condition and valence in regression models for any of the four actors (p > 0.1381 for all).

Finally, we note that across conditions and for both the allocation of responsibility and credit, participants thought the artist was the most accountable, followed by the curator, then the technologist, and finally the crowd. This suggests a robust baseline ordering of the relative importance of the actors for the context of AI-generated art.

## Discussion

No AI acts alone, completely divorced from the influence of humans. Even the artwork *Edmond de Belamy,* which was claimed to be “entirely … realised by an algorithm,” was the result of the creativity, hard work, and decisions of numerous human contributors. When an AI system achieves something great or causes a serious problem, how is responsibility attributed to the humans surrounding it? We explored this question in the domain of AI-generated art. We showed that there is natural heterogeneity in the extent to which individuals perceive AI used to generate art. In addition, perceived anthropomorphicity can be actively manipulated depending on how the AI is described. We also showed that different degrees of anthropomorphicity impact the responsibility attributed to surrounding humans in different ways. Instead of reducing perceived responsibility of all human actors, we instead find that anthropomorphizing the AI system serves to increase responsibility to some actors and decrease responsibility to others. In particular, anthropomorphizing the AI system mitigates the responsibility to the artist, while bolstering the responsibility of the technologist. Critically, this suggests that the responsibility that will be allocated to individuals in the creation of AI art will be dependent on the choice of language and framing used to discuss it. It is important for artists, computer scientists, and the media at large to be aware of the power of their words, and for the public to be discerning in the narratives they consume.

Our results shed light on the responsibility conundrum of the *Edmond de Belamy* case. People allocated the most credit and responsibility to the artist, then the curator, then the technologist, and finally the crowd. These results suggest that although this hierarchy is robust, even the crowd is deemed worthy of a non-trivial amount of responsibility and credit. It seems that our participants think Robbie Barrat, the programmer who created the Github repository that Obvious ostensibly pulled from to create *Edmond de Belamy*, should be given credit for his contribution.

In Study 2, the two conditions we used (Tool and Agent) captured two extremes concerning how AI systems are discussed in the media (see Table 1). The Tool Condition used non-agentic language and described the AI as being manipulated by a human, whereas the Agent Condition used anthropomorphic language and described the AI as taking independent actions. Our vignettes were designed to mirror two general modes of discussing AI in the media ecosystem: agentic/anthropomorphized or tool-like/non-anthropomorphized. Naturally, if an AI is described as having agent-like properties (e.g., making decisions) anthropomorphic language (e.g., that it has desires) will often be used to describe it. Future work should attempt to isolate these variables. Are our results due to the agent-like behavior of the AI, the anthropomorphic language, or both?

Finally, it is important to note that our findings are not straightforwardly prescriptive. We do not intend to make claims about the extent to which various parties *should* be held accountable in the contexts we study. Rather, we are reporting *what participants think* about how accountability should be distributed. Although we do not think that public opinion about accountability should directly translate into policy, public perceptions can be important for policy makers, for instance, to predict public reaction to a policy or to determine how to open public debate on a controversial topic (Rahwan et al., 2019).

### Limitations of the Study

There are several potential limitations to this work. First, as discussed in the discussion, there is the potential confound in Study 2 of agent-like behavior and anthropomorphic language. Future work might attempt to isolate these variables. Second, our studies were run on Amazon's Mechanical Turk. Future work might look at how these effects generalize to other populations. Third, as discussed in the Introduction, our study focuses on a particular method of producing AI-generated artwork. Future work might test the generalizability of our results to other forms of AI-generated art.

### Resource Availability

#### Lead Contact

Further information and requests for resources and reagents should be directed to and will be fulfilled by the Lead Contact, Ziv Epstein (zive@mit.edu).

#### Materials Availability

This study did not generate new unique reagents.

#### Data and Code Availability

The datasets and code generated during this study are available at https://github.com/zivepstein/ai-art-credit.

## Methods

All methods can be found in the accompanying Transparent Methods supplemental file.

## Supplemental Information

Document S1. Transparent Methods and Tables S1 and S2

## Footnotes

Supplemental Information can be found online at https://doi.org/10.1016/j.isci.2020.101515.
