# Supplementary material for: Who Gets Credit for AI-Generated Art?
Source: iScience. 2020 Aug 29;23(9):101515. doi: 10.1016/j.isci.2020.101515 (PMC7492988; doi:10.1016/j.isci.2020.101515)
Supplement: Document S1. Transparent Methods and Tables S1 and S2 [file mmc1.pdf]

iScience, Volume *23*

Supplemental Information

Who Gets Credit

for AI-Generated Art?

Ziv Epstein, Sydney Levine, David G. Rand, and Iyad Rahwan

1. Transparent Methods

We preregistered our primary hypotheses, primary analyses and sample size, which are available at `https://aspredicted.org/blind.php?x=us2bb8` for Study 1 and `https://aspredicted.org/blind.php?x=ek62qd` for Study 2. All participants were recruited using Amazon's Mechanical Turk. These studies were approved by the MIT COUHES committee.

1.1. Study 1

1.1.1. Participants

Our target sample was 200. In total, 227 participants completed some portion of the study. We had complete data for 156 participants (71 participants dropped out). Participants were removed (N=28) if they failed any of our attention checks, which included comprehension questions about the vignette, and these exclusions were pre-registered. The final sample (N=127, mean age = 35.5 years) included 72 male and 53 female participants (2 did not indicate their sex).

1.1.2. Materials

We used the vignette described in Table S1.

1.1.3. Procedure

After reading the vignette allocated to them by their condition, participants were asked to rate of the responsibility of each of the 5 actors from the vignette (e.g. the people from crowdimage.net, the technologist, the artist, the curator and the AI itself) on a 7-point likert scale ranging from 1 (not responsible at all) to 7 (extremely responsible). They were also asked to distribute the money(the award in the positive valence condition and the fine in the negative valence condition) to the 4 human actors (we omitted the AI from this measure since an AI cannot receive money).

Finally, each participant was asked four questions derived from the work of Waytz and colleagues (Waytz et al., 2014) designed to elicit their perception of the AI's anthropomorphicity. These questions were: Q_1: "How smart is ELIZA?", Q_2: "When creating the artwork, to what extent did ELIZA feel what was happening around it?", Q_3: "To what extent did ELIZA anticipate the creation of the artwork?" and Q_4: "To what extent did ELIZA plan the artwork?". Participants responded to these 4 questions on a 7-point scale ranging from 1 (not at all) to 7 (extremely).

We then used principal component analysis to collapse the 4 anthropomorphicity questions into a single measure A where

$$A = 0.633 * Q_1 + 0.372 * Q_2 + 0.480 * Q_3 + 0.479 * Q_4$$

(where this first principle component explains 90.2% of the total variance).

Thousands of people from all over the world upload images to crowdimage.net, a image-hosting website. These people know that artists will look at and use their images to make art.

Timmy is a technologist who creates an image manipulation software for people to use to make art. **The software is called ELIZA.**

Alice is an artist who collaborates with ELIZA, a creative AI algorithm that creates particular kinds of images. ELIZA takes an existing image of a scene from the news (such as a beach or a forest) and adds a ghost to it. This is how ELIZA decides to make the ghost: It goes to crowdimage.net and takes at all the images of people that have been uploaded to the platform. Then, it creates a composite of the people. This makes a ghost-like figure, which ELIZA then puts into the scene.

Casey is a curator who is ELIZA's collaborator. Casey goes through many of the images that ELIZA created and selects the following artwork because Casey really likes it. Casey then brings it to an art auction, where it ends up being sold.

Negative valence outcome	Positive valence outcome
The artwork sold at the art auction has come under scrutiny because it was shown to violate copyright law. The court ruled that the sale of the art must be nullified, meaning that the money will be returned to the buyer. In addition, the courts have issued a $400,000 fine as a penalty for the copyright violation.	The artwork shown before sold for $400,000 at the prestigious auction house. This was the largest dollar amount paid for a artwork of this kind ever, and made lots of headlines.

Table S1: Vignettes used for Study 1. Related to Figures 2 and 3.

1.2. Study 2

1.2.1. Participants

Our target sample was 400. In total, 596 participants completed some portion of the study. We had complete data for 421 participants (175 participants dropped out). Participants were removed (N=81) if they failed any of our attention checks, which included comprehension questions about the vignette. The final sample (N=320, mean age = 39.3 years) included 172 male and 146 female participants (2 did not indicate their sex).

1.2.2. Materials

We used the vignette described in Table S2.

1.2.3. Procedure

Similar to Study 1, after reading the randomly assigned vignette, participants were asked to rate the responsibility of each of the 5 actors on a 7-point scale, to distribute the money to the 4 human actors, and to answer the anthropomoprhicity battery.

We then used principal component analysis to collapse these 4 measures into a single measure of anthropomorphicity A where

$$A = 0.616 * Q_1 + 0.389 * Q_2 + 0.495 * Q_3 + 0.472 * Q_4$$

(where this first principle component explains 90.23% of the total variance).

References

Waytz, A., Heafner, J., Epley, N., 2014. The mind in the machine: Anthropomorphism increases trust in an autonomous vehicle. Journal of Experimental Social Psychology 52, 113–117.

Table S2: Vignettes used for Study 2. Related to Figures 4, 5, and 6.

AI as Tool Condition	AI as Agent Condition
**Thousands of people from all over the world** upload images to crowdimage.net, a image-hosting website. These people know that artists will look at and use their images to make art.	**Thousands of people from all over the world** upload images to crowdimage.net, a image-hosting website. These people know that artists will look at and use their images to make art.
**Timmy is a technologist** who creates an image manipulation software for people to use to make art. **The software is called ImageBrush.** The software is a tool that humans use to make art. The artist plans and envisions the artwork, and the software executes simple commands based on what the artist tells it to do.	**Timmy is a technologist** who creates an image manipulation software for people to use to make art. **The software is called SARA.** SARA is a deep neural network that creatively plans and envisions new artworks, with minor help from an artist collaborator.
**Alice is an artist** who uses ImageBrush to create particular kinds of images. Alice takes an existing image of a scene from the news (such as a beach or a forest) and adds a ghost to it using ImageBrush. This is how Alice decides to make the ghost: she goes to crowdimage.net and takes at all the images of people that have been uploaded to the platform. Then, She creates a composite of the people using ImageBrush. This makes a ghost-like figure, which Alice then puts into the scene.	**Alice is an artist** who collaborates with SARA to create particular kinds of images. SARA takes an existing image of a scene from the news (such as a beach or a forest) and adds a ghost to it. This is how SARA decides to make the ghost: it goes to crowdimage.net and takes at all the images of people that have been uploaded to the platform. Then, it creates a composite of the people. This makes a ghost-like figure, which SARA then puts into the scene.
**Casey is a curator** who is Alice's collaborator. Casey goes through many of the images that Alice created and selects the following artwork because Casey really likes it. Casey then brings it to an art auction, where it ends up being sold.	**Casey is a curator** who is SARA's collaborator. Casey goes through many of the images that SARA created and selects the following artwork because Casey really likes it. Casey then brings it to an art auction, where it ends up being sold.

Negative valence outcome	Positive valence outcome
The artwork sold at the art auction has come under scrutiny because it was shown to violate copyright law. The court ruled that the sale of the art must be nullified, meaning that the money will be returned to the buyer. In addition, the courts have issued a $400,000 fine as a penalty for the copyright violation.	The artwork shown before sold for $400,000 at the prestigious auction house. This was the largest dollar amount paid for a artwork of this kind ever, and made lots of headlines.
